# circRNA_100859 functions as an oncogene in colon cancer by sponging the miR-217-HIF-1α pathway

**DOI:** 10.18632/aging.103438

**Published:** 2020-07-08

**Authors:** Peng Zhou, Wei Xie, Hua-Lin Huang, Rong-Qi Huang, Chao Tian, Hong-Bo Zhu, Ying-Huan Dai, Zhi-Yuan Li

**Affiliations:** 1 Department of Anatomy and Neurobiology, School of Basic Medical Sciences, Central South University, Changsha, Hunan, China; 2 Department of Pathology, The Second Xiangya Hospital, Central South University, Changsha, Hunan, China; 3 CAS Key Laboratory of Regenerative Biology, Guangdong Provincial Key Laboratory of Stem Cell and Regenerative Medicine, Guangzhou Institutes of Biomedicine and Health, Chinese Academy of Sciences, Guangzhou, China; 4 Guangzhou Regenerative Medicine and Health Guangdong Laboratory, Guangzhou, China; 5 Hepatology Unit and Department of Infectious Diseases, Nanfang Hospital, Southern Medical University, Guangzhou, China; 6 School of Life Sciences, University of Science and Technology of China, Hefei, China; 7 GZMU-GIBH Joint School of Life Sciences, Guangzhou Medical University, Beijing, China; 8 University of Chinese Academy of Sciences, Beijing, China

**Keywords:** colon cancer, circRNA_100859, microRNA-217, HIF-1α, biomarker

## Abstract

Circular RNAs (circRNAs) play an important role in cancer development and progression by regulating gene expression. The present study aimed to investigate the function of circRNA_100859 in colon cancer. circRNA expression profiles from a human circRNAs chip were analyzed. The effects of circRNA_100859 on cell proliferation and apoptosis were assessed *in vitro* and interactions between circRNA_100859 and its micro (mi)RNA and target genes were analyzed. The diagnostic and prognostic significance of circRNA_100859 was also investigated. It was identified that circRNA_100859 was overexpressed in colon cancer tissues and promoted cell proliferation and inhibited cell apoptosis. Additionally, bioinformatics and a dual-luciferase reporter assay confirmed that circRNA_100859 acted as a miR-217 sponge, and miR-217 directly targeted hypoxia-inducible factor (HIF)-1α. Rescue assays demonstrated that HIF-1α protein and mRNA expression levels and cell proliferation were regulated by the circRNA_100859/miR-217 axis (*P*<0.05). Furthermore, statistical analysis showed that the circRNA_100859-miR-217-HIF-1α axis was associated with Tumor-Node-Metastasis (TNM) stage, histological grade, and KRAS mutations, and also showed high diagnostic and prognostic value for patients with colon cancer (*P*<0.05). Therefore, it was concluded that circRNA_100859 functions as an oncogene in colon cancer by sponging the miR-217-HIF-1α pathway. In addition, the circRNA_100859-miR-217-HIF-1α axis may serve as a novel diagnostic and prognostic biomarker for patients with colon cancer.

## INTRODUCTION

Colon cancer is derived from intestinal epithelium and is one of the most common gastrointestinal malignancies, and the incidence of the disease has rapidly increased in recent years [1]. The tumorigenesis and metastasis of colon cancer is a complicated process with many steps concerning the activation of oncogenes and inactivation of anti-oncogenes [2, 3]. Although comprehensive therapies have been used, the prognosis of colon cancer is still poor, which may be due to the lack of early diagnosis and effective targeted therapy agents [4]. Therefore, efficient diagnosis and therapeutic approaches are important for colon cancer research.

non-coding RNA (ncRNA) plays an important role in the regulation of gene expression levels, RNA shearing and modification but does not encode proteins [5]. microRNAs (miRNAs/miRs) are an important class of endogenous single-stranded ncRNAs and function as sequence-specific negative regulators in post-transcriptional gene silencing by base-pairing with target mRNAs, which leads to mRNA cleavage or translational repression [6]. circRNAs are a type of endogenous ncRNAs characterized by covalently closed-loop structures and regulate gene expression in eukaryotic cells [7]. Many studies [8, 9] have highlighted that circRNAs are extensively involved in various physiological processes and abnormal expression and regulation of circRNAs are closely related to carcinogenesis, progression, and metastasis of tumors. circRNAs can be divided into three subtypes according to the sequence formation and combination [10]: exon-derived, intron-derived, and exonic-intronic. Current studies [10, 11] argue that circRNA functions as a miRNA sponge suppressing miRNA expression levels and function, and ultimately modulates gene transcription at the post-transcriptional level. Also, some circRNAs may play crucial roles in gene transcription and protein synthesis, but the precise mechanism of these functions remains unclear and requires further study.

In the present study, circRNA expression profiles were screened and it was found that circRNA_100859 was overexpressed in colon cancer tissues, and circRNA_100859 promoted colon cancer progression by sponging miR-217 to upregulate hypoxia-inducible factor (HIF)-1α expression levels. This indicated that the circRNA_100859-miR-217-HIF-1α axis may have value in diagnosis and prognosis prediction for patients with colon cancer.

## RESULTS

### Different circRNA expression profiles of colon cancer

The differential circRNA expression profiles of three paired colon cancer and adjacent tissues were estimated using the Arraystar Human circRNAs chip. Scatterplots (Supplemental Figure 1A) and volcano plots (Supplemental Figure 1B) demonstrated the variation of differentially expressed circRNAs between colon cancer and adjacent tissues. The circRNAs chip signals diagram based on the differentially expressed circRNAs were obtained by using an Axon Gene Pix 4000B Chip scanner (Supplemental Figure 1C).

### circRNA_100859 is significantly overexpressed in colon cancer

**Figure 1:**
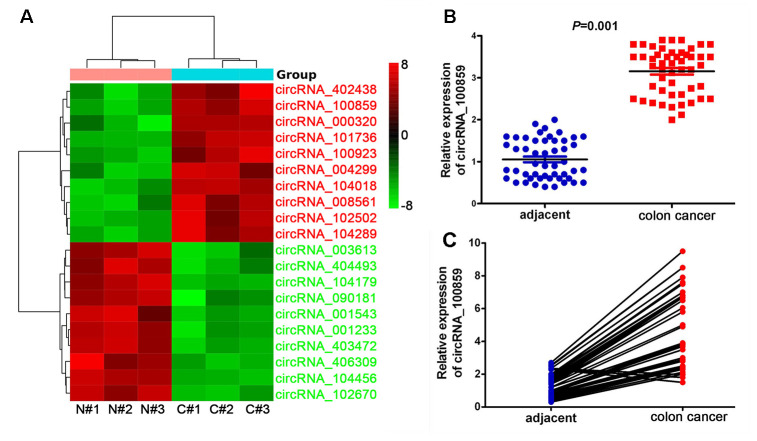
**circRNA_100859 is significantly overexpressed in colon cancer and verified by RT-qPCR (n=3).** (**A**) The heat mapping revealed the top ten significantly increased and decreased circRNAs. (**B**) RT-qPCR assay verifies that it significantly over-expressed in colon cancer tissues (n=50), compared with adjacent non-cancer tissues. (**C**) Matched pair analysis showed the expression of circRNA_100859 in colon cancer tissues was mostly higher than corresponding adjacent non-cancer tissues. N: adjacent non-cancer tissues, C: colon cancer tissues.

After normalization and data analysis, there were 103 differentially expressed circRNAs, including 32 up-regulated by 2-fold and 71 down-regulated by 2-fold in colon cancer tissues compared with adjacent normal tissues. Heat mapping (Figure 1A) revealed the top 10 significantly up and down-regulated circRNAs in colon cancer compared with adjacent normal tissues. Full details these top 10 differentially expressed circRNAs are listed in Table 1. Of the 20 differentially expressed circRNAs, after filtering out circRNAs with low raw intensity, it was found that circRNA_100859 was significantly overexpressed by 6.98-fold in colon cancer tissue in high raw intensity samples. RT-qPCR was used to verify the circRNAs chip analysis results. The results demonstrated that circRNA_100859 was significantly overexpressed in colon cancer tissues (n=50) (Figure 1B, *P*<0.05), compared with adjacent tissues, and matched pair analysis showed that the expression levels of circRNA_100859 in colon cancer tissues were higher than in corresponding adjacent non-cancer tissues (Figure 1C, *P*<0.05).

**Table 1:** The top 10 significantly up-and down-regulated circRNAs.

**circRNA**	**P-value**	**FDR**	**FC (abs)**	**Regulation**	**source**	**chrom**	**type**	**GeneSymbol**
hsa_circRNA_402438	0.036410256	0.541205763	6.9848956	up	25242744	chr2	exonic	ORC2
hsa_circRNA_100859	0.011448038	0.541205763	6.6872112	up	circBase	chr11	exonic	KDM2A
hsa_circRNA_000320	0.037806042	0.541205763	6.056733	up	circBase	chr11	intronic	AHNAK
hsa_circRNA_101736	0.028113005	0.541205763	5.9997955	up	circBase	chr16	exonic	SMG1
hsa_circRNA_100923	0.028496393	0.541205763	5.0672873	up	circBase	chr11	exonic	PICALM
hsa_circRNA_004299	0.036327512	0.541205763	5.0554866	up	circBase	chr7	exonic	ARPC1B
hsa_circRNA_104018	0.014580554	0.541205763	5.0397681	up	circBase	chr5	exonic	SFXN1
hsa_circRNA_008561	0.046482742	0.541205763	4.5019564	up	circBase	chr22	exonic	TCF20
hsa_circRNA_102502	0.010099194	0.541205763	4.4760374	up	circBase	chr19	exonic	URI1
hsa_circRNA_104289	0.040986664	0.541205763	4.469468	up	circBase	chr7	exonic	EIF3B
hsa_circRNA_003613	0.032246337	0.541205763	6.0363774	down	circBase	chr1	exonic	NRD1
hsa_circRNA_404493	0.007203568	0.541205763	5.5870737	down	25070500	chr1	sense overlapping	C1orf50
hsa_circRNA_104179	0.029554524	0.541205763	5.5658284	down	circBase	chr6	exonic	ZUFSP
hsa_circRNA_090181	0.038800405	0.541205763	5.0924081	down	circBase	chrX	exonic	DMD
hsa_circRNA_001543	0.047464488	0.541205763	5.0328232	down	circBase	chr7	sense overlapping	RSBN1L
hsa_circRNA_001233	0.031753546	0.541205763	5.0098082	down	circBase	chr12	antisense	EP400NL
hsa_circRNA_403472	0.036627096	0.541205763	4.5210286	down	25242744	chr5	exonic	NR3C1
hsa_circRNA_406309	0.01797211	0.541205763	4.1653952	down	25070500	chr3	intronic	CMSS1
hsa_circRNA_104456	0.026157055	0.541205763	3.0142349	down	circBase	chr7	exonic	PUS7
hsa_circRNA_102670	0.012829811	0.541205763	2.6244007	down	circBase	chr2	exonic	BIRC6

### Cells transfected successfully

**Figure 2:**
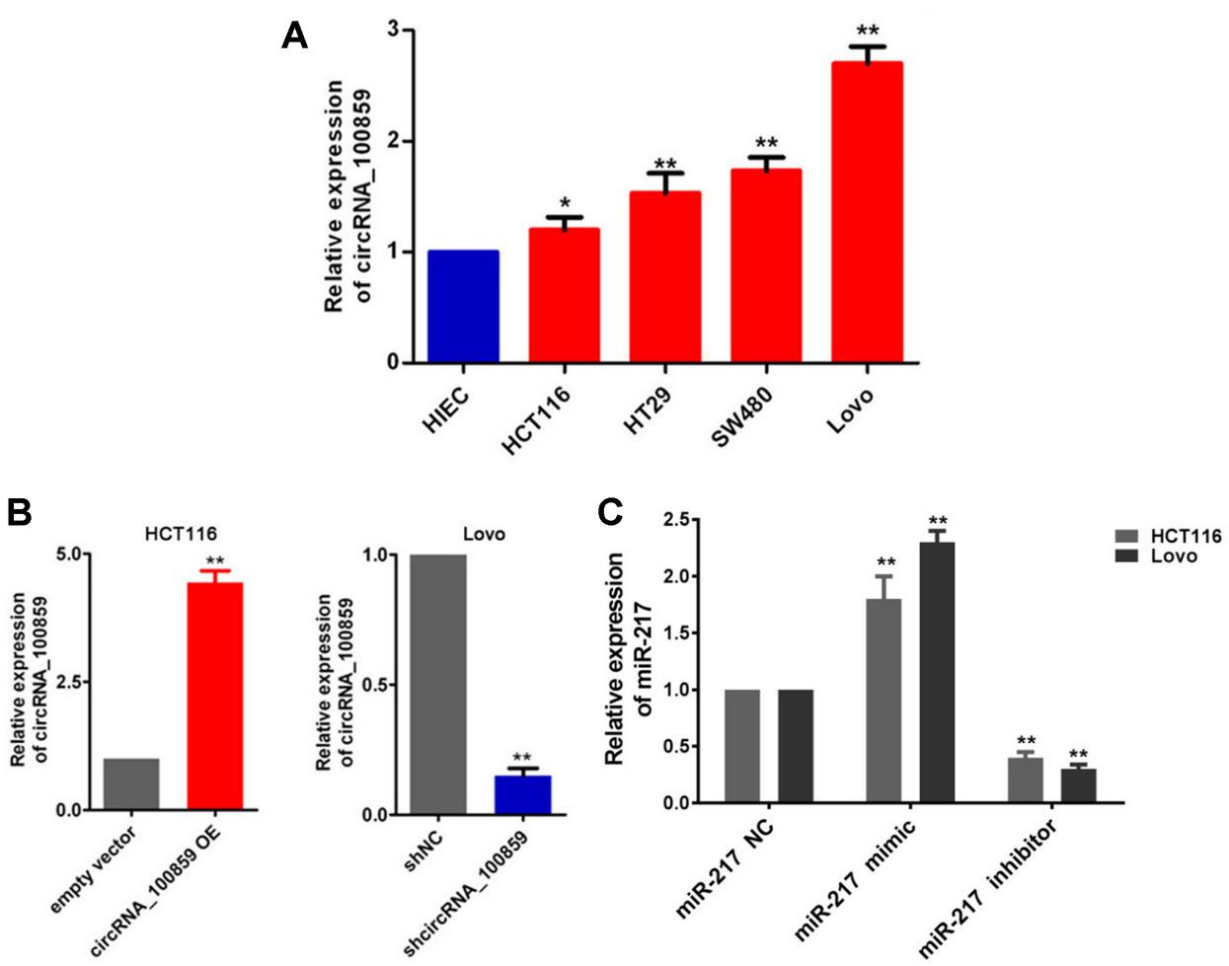
**Cell transfected successfully.** (**A**) RT-qPCR assay showed that circRNA _ 100859 was up-regulated in Lovo, HT29, SW480, HCT116 cells, *P < 0.05, **P < 0.01 versus HIEC cells. (**B**) RT-qPCR assay showed that circRNA_100859 was increased in HCT116 cells, and decreased in Lovo cells. (**C**) RT-qPCR assay demonstrated that miR-217 expression levels were significantly increased in the mimic group, and significantly reduced in inhibitor group. *P < 0.05, **P < 0.01 versus corresponding NC group.

In the present study, HCT116, HT29, SW480, Lovo cells and HIEC cells were used. RT-qPCR analysis demonstrated that circRNA_100859 was most highly expressed in Lovo cells, followed by HT29 and SW480 cells, whilst the lowest expression levels of circRNA_100859 were observed in HCT116 cells compare with HIEC cells (Figure 2A, *P*<0.05). The plasmid overexpressing circRNA_100859 was transfected into HCT116 cells to upregulate circRNA_100859 expression levels, while shRNA was transfected into Lovo cells to silence circRNA_100859 expression. RT-qPCR results showed that the expression levels of circRNA_100859 were significantly increased in the circRNA_100859 overexpressing group, compared with the empty vector group, and dramatically decreased in the sh-circRNA_100859 group, compared with the sh-NC group (Figure 2B, *P*<0.05). Also, the miR-217 mimic, inhibitor, and NC were transfected into Lovo and HCT116 cells. RT-qPCR assay results confirmed that miR-217 expression levels were significantly increased in the mimic group, and significantly reduced in inhibitor group, compared with the NC group (Figure 2C, *P*<0.05).

### circRNA_100859 promotes cell proliferation and inhibit cell apoptosis

**Figure 3:**
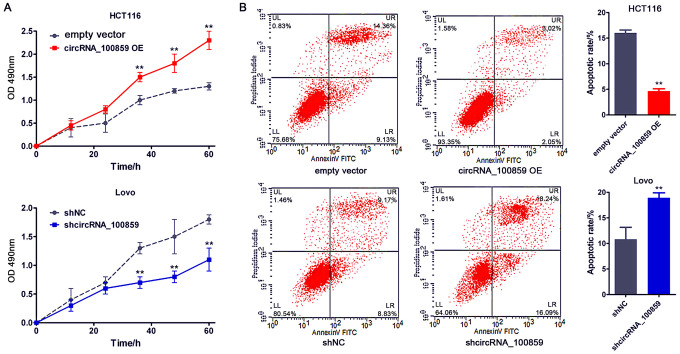
**circRNA_100859 can promote cell proliferation and inhibit cell apoptosis. (n=3).** (**A**) MTT assay; (**B**) Flow Cytometry assay. ^*^P < 0.05, ^**^P < 0.01 versus corresponding NC group.

MTT and flow cytometry assays were used to assess the role of circRNA_100859 in cell proliferation and apoptosis *in vitro*. The MTT assay indicated that circRNA_ 100859 overexpression significantly promoted cell proliferation in HCT116 cells, while circRNA_100859 silencing dramatically inhibited cell proliferation in Lovo cells (Figure 3A, *P*<0.05). Conversely, for cell apoptosis, the flow cytometry assay demonstrated that circRNA_100859 overexpression inhibited the rate of apoptosis, while circRNA_100859 silencing increased the rate of apoptosis (Figure 3B, *P*<0.05). Together, the aforementioned results demonstrated that circRNA_100859 overexpression promoted cell proliferation and inhibited cell apoptosis, and that circRNA_100859 silencing can inhibit cell proliferation and promote apoptosis, indicating that circRNA_100859 has an oncogenic role in colon cancer procession.

### circRNA_100859 acts as a competing endogenous (ce)RNA to sponge miR-217

**Figure 4:**
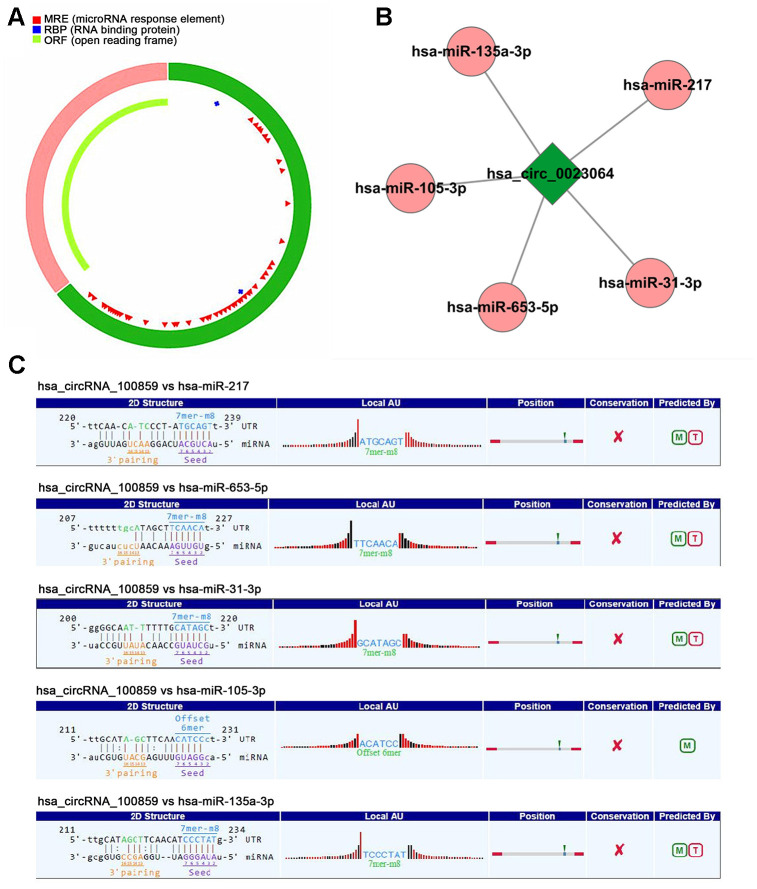
**circRNA-miRNA interaction** (**A**) The fundamental structure modes of circRNA_100859 predicted by CSCD. (**B**) 5 potential target miRNAs of circRNA_100859. (**C**) The interaction sequences of circRNA_100859 to miRNAs.

**Figure 5:**
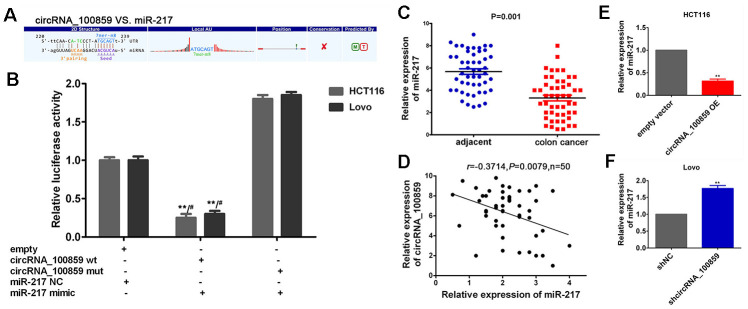
**circRNA_100859 acts as a ceRNA to sponge miR-217.** (**A**) The potential binding sequences of circRNA_100859 to miR-217. (**B**) The relative luciferase activities were estimated after co-transfection with circRNA_100859-wt or mut and miR-217 mimics or miR-217 NC were analyzed. ^**^P<0.01 vs. empty vector+NC. ^#^*P*>0.05 vs. mut+mimic. (**C**) miR-217 was dramatically down-regulated in colon cancer tissues (n=50), ^*^P < 0.05, ^**^P < 0.01 versus adjacent non-cancer tissues. (**D**) Pearson's analysis suggested that the negative correlation between circRNA_100859 and miR-217. (**E**, **F**) RT-qPCR demonstrated that circRNA_100859 overexpression inhibited miR-217 expression, while circRNA_100859 silencing increased miR-217 expression. ^*^P < 0.05, ^**^P < 0.01 versus corresponding NC group.

The fundamental structure of circRNA_100859 were predicted using Cancer-Specific circRNA (http://gb.whu.edu.cn/CSCD) and shown in Figure 4A. circRNA can function as a miRNA sponge to suppress miRNA expression. In order to elucidate the interaction between circRNA_100859 and its target miRNAs, the miRanda database was used (http://www.ebi.ac.uk/enright-srv/microcosiTi/htdo/targets/v5) to predict the circRNA _100859 binding sites on target miRNAs. There were 5 potential target miRNAs predicted, and miR-217 showed the highest context score (Figure 4B, 4C, *P*<0.05). miR-217 was selected (Figure 5A) for further analysis. To determine whether circRNA_100859 directly targeted miR-217, a dual-luciferase reporter assay was performed to verify the MRE-based circRNA_100859-miR-217 interaction. psiCHECK2-circRNA_100859-wt and mut type plasmids were constructed, and luciferase activity was estimated after co-transfection with miR-217 mimic and miR-217 NC. The results demonstrated that the miR-217 mimic significantly inhibited the activity of circRNA_100859-wt in HCT116 and Lovo cells compared with the miR-217 NC (Figure 5B, *P*<0.05). Additionally, the expression levels of miR-217 in colon cancer tissues were determined using RT-qPCR. The results indicated that miR-217 was dramatically down-regulated in colorectal cancer tissues (n=50) (Figure 5C, *P*<0.05), and Pearson’s analysis showed that was a negative correlation between circRNA_100859 and miR-217 (Figure 5D, *P*<0.05). Furthermore, it was found that circRNA_100859 overexpression inhibited miR-217 expression, while circRNA_100859 silencing increased miR-217 expression levels (Figure 5E, 5F, *P*<0.05). In brief, the aforementioned results demonstrated that circRNA_100859 acts as a miRNA sponge for miR-217 in colon cancer.

### HIF-1α is directly targeted by miR-217

**Figure 6:**
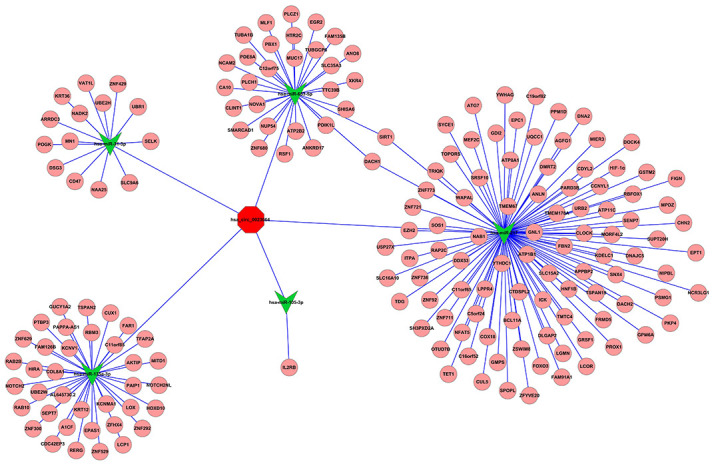
**circRNA-miRNA-mRNA network.** Yellow color represents circRNA_100859, the red color and light-blue color represent miRNA and mRNA, respectively.

**Figure 7:**
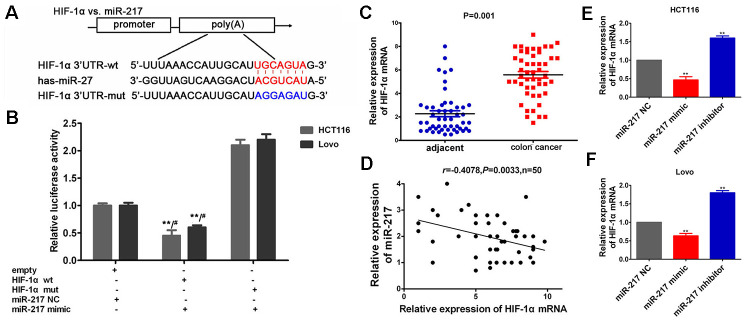
**HIF-1α was directly targeted by miR-217**. (**A**) The potential binding sequences of HIF-1α to miR-217. (**B**) T The relative luciferase activities were estimated after co-transfection with HIF-1α-wt or mut and miR-217 mimics or miR-217 NC. **P<0.01 vs. empty vector+NC. #P>0.05 vs. mut+mimic. (**C**) HIF-1αwas significantly over-expressed in colon cancer tissues (n=50), *P < 0.05, **P < 0.01 versus adjacent noncancer tissues. (D) Pearson's analysis suggested that the negative correlation between HIF-1α and miR-217. (E, F) RT-qPCR demonstrated that HIF-1α mRNA expression were significantly decreased in miR-217 mimic group, and dramatically increased in miR-217 inhibitor group. *P < 0.05, **P < 0.01 versus miR-217 NC group.

To further elucidate the interaction between miR-217 and potential targeting genes, the target genes of miR-217 were predicted using miRanda version 5 (http://www.ebi.ac.uk/enright-srv/microcosiTi/htdo/targets/v5), TargetScan (http://www.targetscan.org), and mibase (http://pictar.mdc-berlin.de/). The circRNA-miRNA-mRNA network was predicted using Cytoscape software (version 3.6.1:http://cytoscape.org/) and HIF-1α had relatively high target score (Figure 6). To assess the targeting regulatory relationship between HIF-1α and miR-217. The miR-217 binding site in the HIF-1α 3’untranslated region is shown in Figure 7A and the dual-luciferase reporter assay demonstrated that the miR-217 mimic significantly inhibited the activity of HIF-1α-wt in HCT116 and Lovo cells compared with the miR-217 NC (Figure 7B, *P*<0.05). Additionally, RT-qPCR demonstrated that HIF-1α mRNA was markedly overexpressed in colon cancer tissues (n=50) (Figure 7C, P<0.05), and Pearson’s analysis indicated that there was negative correlation between miR-217 and HIF-1α mRNA expression levels (Figure 7D, P<0.05). Furthermore, HIF-1α mRNA expression levels were significantly decreased in the miR-217 mimic group and dramatically increased in the miR-217 inhibitor group compared with the miR-217 NC group both in HCT116 and Lovo cells (Figure 7E, 7F, P<0.05). These results suggested that HIF-1α was directly targeted by miR-217 in colon cancer.

### Rescue assays

**Figure 8:**
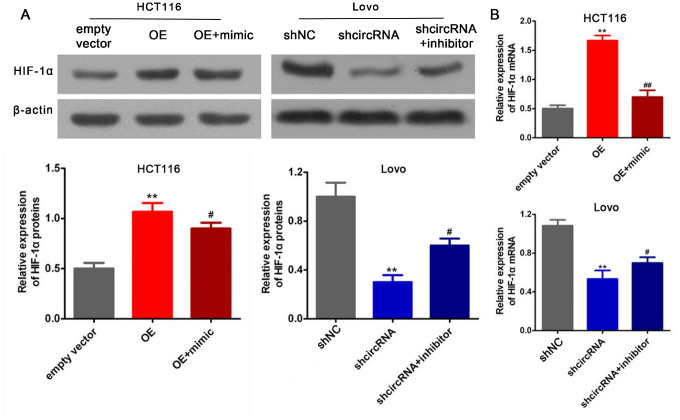
**circRNA_100859 can directly sponge to miR-217 to further target HIF-1α.** (**A**) HIF-1α protein expression. (**B**) HIF-1α mRNA expression. ^*^P < 0.05, ^**^P < 0.01 versus corresponding NC group.

**Figure 9:**
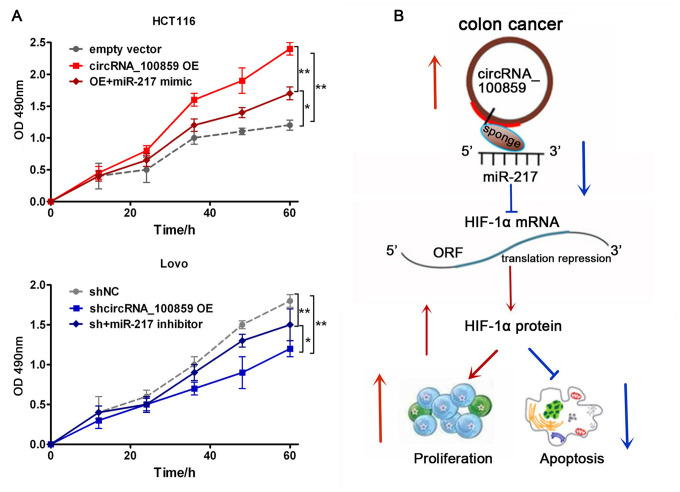
**Rescue assays.** (**A**) MTT assay showed the effects of circRNA_141539/miR-4469 axis on cell proliferation, ^*^P < 0.05, ^**^P < 0.01 versus corresponding NC group. (**B**) Schematic diagram of circRNA_100859-miR-217- HIF-1α axis in colon cancer procession.

The aforementioned studies showed that circRNA_100859 acts as a ceRNA to sponge miR-217 and HIF-1α by directly targeted miR-217. Furthermore, in order to determine the interaction between the circRNA_100859-miR-217 axis and HIF-1α, and the roles of the circRNA_100859-miR-217-HIF-1α axis in colon cancer progression, rescue assays were performed. HIF-1α expression levels and cell proliferation were detected after co-transfection with the circRNA_100859 overexpressing plasmid and miR-217 mimic in HCT116 cells, and co-transfection with sh-circRNA_100859 and miR-217 inhibitor into Lovo cells. The results showed that circRNA_100859 overexpression increased HIF-1α protein and mRNA expression levels, but this effect was reversed by the miR-217 mimic, while circRNA_100859 silencing inhibited HIF-1α protein and mRNA expression, but this effect was reversed by the miR-217 inhibitor (Figure 8A, 8B, P<0.05). Furthermore, the miR-217 mimic rescued proliferation in HCT116 cells with circRNA_100859 overexpression, and the miR-217 inhibitor rescued proliferation in Lovo cells with circRNA_100859 silencing (Figure 9A, P<0.05). In short, the above results indicated that HIF-1α expression levels and cell proliferation were regulated by the circRNA_100859-miR-217 axis, and that the circRNA_100859-miR-217-HIF-1α axis contributed to colon cancer progression. In brief, as illustrated in Figure 9B, these results demonstrated that circRNA_100859 can directly sponge miR-217 to target HIF-1α, contributing to colon cancer progression.

### Diagnostic and prognostic value of the circRNA_100859-miR-217-HIF-1α axis in colon cancer

**Figure 10:**
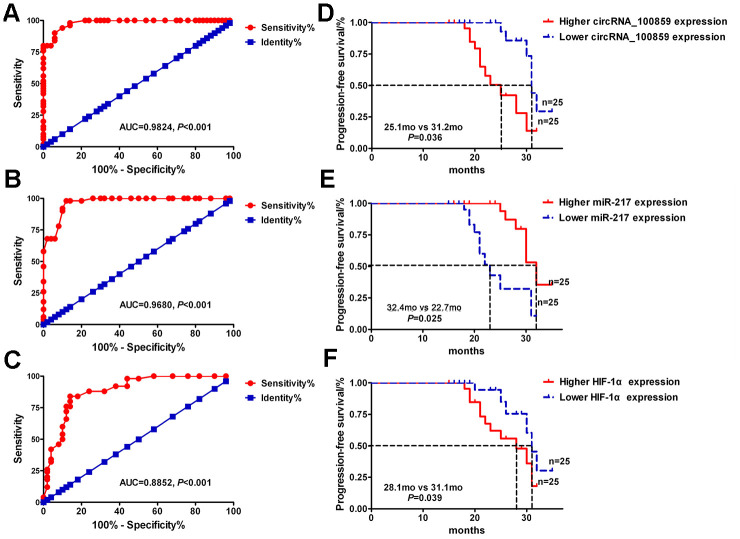
**Diagnostic and Prognostic Value of circRNA_100859-miR-217-HIF-1α axis in colon cancer.** ROC curve for (**A**) circRNA_100859, (**B**) miR-217, and (**C**) HIF-1α expression. Kaplan-Meier survival curve of (**D**) higher and lower circRNA_100859, (**E**) miR-217, and (**F**) HIF-1α expression in colon cancer patients..

**Figure 11:**
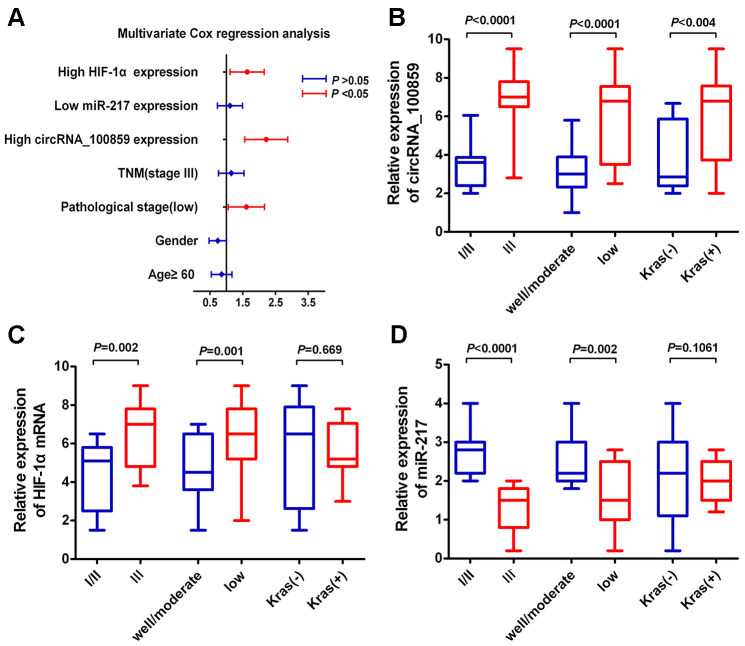
**Relationship between circRNA_100859-miR-217-HIF-1α axis and clinic pathological features.** (**A**) Multivariate Cox regression analysis. (**B**) Box Plots for analysis circRNA_100859 expression, (**C**) Box Plots analysis for miR-217 expression. (**D**) Box Plots analysis for HIF-1α expression.

Receiver operating curve analysis was used to explore the potential diagnostic value of the circRNA_100859- miR-217-HIF-1α axis in patients with colon cancer. The results demonstrated that components the circRNA_100859-miR-217-HIF-1α axis showed high diagnostic efficiency for patients with colon cancer with

area under the curve (AUC) =0.9865, 0.9680, and 0.8825, respectively (Figure 10A-C, *P*<0.05). Also, all 50 colon cancer cases were divided into high and low circRNA_100859, miR-217, and HIF-1α expression groups by using the median circRNA_100859, miR-217, and HIF-1α expression values, respectively. Kaplan-Meier survival analysis indicated that high circRNA_100859 and HIF-1α expression levels were associated with shorter progression-free survival (PFS) (median (m) PFS, 21.5 vs 31.2 months, *P*=0.036; 28.1 months vs 31.1 months, *P*=0.039; Figure 10B), and lower miR-217 expression levels were correlated with shorter PFS (mPFS, 32.4 vs 22.7 months, *P*=0.025, Figure 10D-F). Furthermore, multivariate Cox regression analysis revealed that high circRNA_100859, low miR-217, high HIF-1α expression levels as well as histological grade were a poor prognostic factors for colon cancer (Figure 11A). These results demonstrated that the circRNA_100859-miR-217-HIF-1α axis showed high diagnostic and prognostic value in patients with colon cancer.

### Relationship with clinicopathological features

The association between the circRNA_100859-miR-217-HIF-1α axis and clinicopathological features were analyzed using Chi-square tests. The results indicated that stage III and poorly differentiated tissues showed higher circRNA_100859 and higher HIF-1α expression levels compared with stage I/II and well/moderate differentiated tissues (Figure 11B, 11C). On the contrary, stage III and poorly differentiated tissues showed lower miR-217 expression levels (Figure 11D). Also, circRNA_100859 expression levels were positively associated with *KRAS* mutations, but miR-217 and HIF-1α expression levels were not correlated with *KRAS* mutations.

## DISCUSSION

Previous studies have proved that the development of colon cancer is a complicated process, involving the activation of multiple oncogenes and inactivation of tumor suppressor genes, and is also often accompanied by epigenetic modifications. ncRNA is an important component of epigenetic regulation, including regulation of gene expression levels and participating in a variety of biological processes [12, 13]. Also, unlike linear ncRNA, such as miRNA or long non-coding RNA, circRNAs are a special type of endogenous ncRNAs characterized by a covalently closed loop without 5’ caps and 3’ tails and are highly conserved and stably expressed in eukaryotic cells. Recent research [14, 15] indicated that abnormal expression and regulation of circRNAs are involved in the occurrence and development of a variety of tumors. Li et al. [16] reported that circRNA_0002483 can enhance Taxol sensitivity of non-small cell lung cancer cells by targeting miR-182-5p. Additionally, Liu et al. [17] reported that circRNA_5692 inhibits hepatocellular carcinoma progression by sponging miR-328-5p to enhance DAB2IP expression levels. Furthermore, Wang et al. [18] reported that circRNA_LMTK2 acts as a miR-150-5p sponge and promotes proliferation and metastasis in gastric cancer. However, research investigating the function of circRNAs in colon cancer is limited.

In the present study, different circRNA expression profiles of colon cancer were established using a circRNA chip and it was demonstrated that circRNA_100859 was significantly overexpressed in colon cancer tissues using RT-qPCR. In vitro assays demonstrated that circRNA_100859 promoted cell proliferation and inhibited cell apoptosis, suggesting that circRNA_100859 functions an oncogene and may serve as a novel therapeutic target in colon cancer treatment. From the chip data, circRNA_100859 was exonic and located on chromosome 11: 66985201-66986874. Using the circNet database, miR-217 was a predicted target of circRNA_100859 and this was confirmed using a dual-luciferase reporter assay. In addition, miR-217 was down-regulated and negatively correlated with circRNA_100859, which suggested that circRNA_100859 acts as a miR-217 sponge in colon cancer. miR-217 is located on chromosome 2p16.1 [19], and a previous study [20] reported that miR-217 functions as a tumor suppressor and is a useful diagnostic biomarker and prognosis predictor for several types of cancer. The present findings indicated that the circRNA_100859- miR-217 axis is involved in colon cancer progression.

Additionally, in order to further determine the mechanisms responsible for the oncogenic role of circRNA_100859-miR-217 axis, target genes and biological processes were explored. Prediction from bioinformatics tools combined with luciferase reporter assays demonstrated that miR-217 directly targeted the *HIF-1α* gene and inhibited its expression. Rescue assays further suggested that HIF-1α expression levels and cell proliferation was regulated by the circRNA_100859- miR-217 axis. These results indicated that the circRNA_100859-miR-217-HIF-1α axis contributes to colon cancer progression. Hypoxia is the most common and obvious neoplastic microenvironment in solid tumors and occurs due to the rapid proliferation of tumor cells [21, 22]. HIF-1α is a nuclear protein with transcriptional activity in the hypoxic environment [23] and HIF-1α can activate downstream genes participating in crucial aspects of cancer biology, including angiogenesis, cell survival, glucose metabolism and invasion [24, 25]. In the present study, it was speculated that the circRNA_100859-miR-217-HIF-1α axis may promote colon cancer progression and that HIF-1α may be a potentially novel therapeutic target for colon cancer.

Furthermore, circRNA-miRNA-mRNA axis components may function as potential biomarkers for disease diagnosis and treatment. The diagnostic and prognostic significance of the circRNA_100859-miR-217-HIF-1α axis in colon cancer were analyzed in the present study. Statistical analysis showed that the circRNA_ 100859- miR-217-HIF-1α axis was associated with Tumor-Node-Metastasis stage, histological grade, showing high diagnostic and prognostic value for patients with colon cancer. Notably, it was found that circRNA_100859 levels were positively associated with *KRAS* mutations, but miR-217 and HIF-1α expression levels were not correlated with *KRAS* mutations. This may be a key part of future research.

However, there are several limitations of the present study. Firstly, no *in vivo* experiments were performed. Secondly, the biological processes responsible for the oncogenic role of circRNA_100859 need further investigation. Furthermore, only 50 patients were enrolled, therefore the sample size was relatively small and the results of the present study require further large-scale clinical validation.

## CONCLUSION

The present study identified that circRNA_100859 was significantly overexpressed in colon cancer tissues for the first time, to the best of our knowledge. It was also demonstrated that circRNA_100859 functions as an oncogene in colon cancer by sponging the miR-217-HIF-1α pathway. Furthermore, the components of the circRNA_100859-miR-217-HIF-1α axis may serve as a novel diagnostic and prognostic biomarkers for patients with colon cancer, although further exploration and large-scale clinical validation is needed.

## MATERIALS AND METHODS

### Materials

### Tissues and cells

Fifty samples of pathologically diagnosed colon cancer with paired adjacent normal tissues were obtained from the Department of Pathology, the Second Xiangya Hospital. All participants gave written informed consent and the study was approved by The Institutional Ethical Review Board. None of cases received chemoradiotherapy prior to operation. All samples were stored at -80 °C refrigerator after operation. After surgery, all patients received standard treatment according to National Comprehensive Cancer Network (NCCN) guidelines, and all patients follow up ranged 15-35 months, the median time of follow-up was 23 months, the last follow-up date was 12/25/2019. Human colon cancer cell lines HCT116, HT29, SW480, Lovo and normal human intestinal epithelial (HIEC) cell lines were bought from Cell Resource Center, Shanghai Institutes for Biological Sciences. The cells after resuscitation were cultured in high glucose DMEM medium, containing 10% fetal bovine serum and incubated at a constant incubator at 37°C, 5% CO_2_. All the cell lines used in the experiments were approved by the institutional ethical review board.

### Reagents

RPMI1640, fetal bovine serum and trypsase all were bought from Sigma Co.Ltd (USA). RNeasy Mini Kit were purchased from Hilden Co.Ltd (Germany). Arraystar Human circRNAs chip were purchased from Rockville Co. Ltd(USA). TRIzol reagent, MTT, DMSO, crystal violet, PCR reverse transcription kit were bought from Invitrogen Co.Ltd (USA). Annexin V/PI cell apoptosis kits were purchased from Sigma Co.Ltd (USA). Rabbit anti-HIF-1α antibody (1:1000 dilution, catalog number: ab203848), and Goat Anti-Rabbit IgG H&L (HRP) (1:1000, catalog number: ab205718) were purchased from Abcam Co.Ltd (USA).

### circRNA chip detection

The total RNA of three paired colon cancer and adjacent normal tissues were obtained using a RNeasy Mini kit (Qiagen, Hilden, Germany), and RNA quality and quantity were evaluated using a ND-2000 nanodrop spectrophotometer. Then, linear RNAs were removed and circRNAs were amplified. Different circRNA expression profiles were evaluated using the ArrayStar Human circRNs chip (8×15 K, Arraystar, Rockville, MD, USA). circRNAs demonstrating *P*-values < 0.05 had significant differential expression. The log2-ratio was used for quantile normalization. circRNA detection was performed using Shanghai Genechem Co. Ltd (Chinese).

### Reverse transcription-quantitative PCR (RT-qPCR)

Total RNA was extracted from paired tissues using TRIzol® reagent (Invitrogen). cDNA was synthesized using a RT kit. qPCR was performed using the SYBR Green system. circRNAs and mRNA relative expression levels were estimated using the 2^−ΔΔCt^ method (ΔCt=Ct^target^ -Ct^U6^). The Forward (F) and reverse (R) primers sequence for qRT-PCR were as follows: circRNA_100859: Forward: 5′-TATGCAGTTAAAAATATACA-3′, Reverse: 5′-GATCGGACACGGGTCTT-3′; miR-217: Forward: 5′-ACCTCCAGCTGGGAGGTTAGTCAAGGACTA-3′, Reverse: 5′-TGGTGTCGTGGAGTCG-3′; HIF-1α: Forward: 5′-TGATTGCATCTCCATCTCCTACC-3′, Reverse: 5′-GACTCAAAGCGACAGATAACACG-3′; U6: Forward: 5′-CGCTTCGGCAGCACATATACTAAAATTGGAAC-3′, Reverse: 5′-GCTTCACGAATTTGCGTGTCATCCTTGC-3′.

### Cell transfection

The full length of circRNA_100859 (circRNA_100859 overexpression) was cloned into pcDNA3.1 vector, and the small hairpin (sh)RNA sequence targeting circRNA_100859 (sh-circRNA_100859) was inserted into a lentiviral vector using Shanghai Genechem Co. Ltd (Chinese). All vector were labeled with a green fluorescent protein (GFP). The sequence of circRNA_100859 OE and si-circRNA_100859 were 5′-TCCCTATGCAGTTAAAAAT-3′, 5′-ATACAACATTGAAGATCG-3′, respectively. The concentration of plasmid were 50 nM. The miR-217 mimic and miR-217 inhibitor were also constructed by Shanghai Genechem Co. Ltd (Chinese). The sequence of miR-217 mimics, inhibitors and Negative control were 5′-UACUGCAUCAGGAACUGAUUGGAU-3′, 5′-ATCCAATCAGTTCCTGATGCAGTA-3′, 5′-CUCAAGGAUGCGUUAAGGUUACUA-3′, respectively.

Transfection was performed using Lipofectamine®^TM^ 3000 transfection reagent (Invitrogen). A pcDNA3.1 empty vector, sh-negative control (NC) and miR-217 NC were used as NCs. After 24 h of transfection, the transfection efficiency was observed under a fluorescence microscope and verified using RT-qPCR.

### MTT assay

Transfected cells in the logarithmic growth phase were seeded into 96-well culture plates and cultured for 0, 12, 24, 48, and 72 h. Then, 20 μl MTT regent was added to each well for 4 h. In turn, 100 μl DMSO was added into each well for 30 min. The absorbance was assessed at optical density 490 nm using a micro plate reader.

### Flow cytometry

Transfected cells in logarithmic growth phase were seeded into 96-well culture plates, and cultured for 48 h. Then, 5 μl Annexin V-FITC and 5 μl propidium iodide were added into each well for 15 min and 30 min, respectively. Apoptotic cells were evaluated using flow cytometry (Attune NxT, ThermoFisher).

### Luciferase assay

circRNA_100859 sequence [wild-type (wt) and mutant (mut)] and full-length HIF-1α (wt and mut) were amplified using PCR and cloned into the dual-luciferase reporter pmirGLO vector and co-transfected with the miR-217 mimic or miR-217 NC into HCT116 and Lovo cells. After transfection for 48 h, luciferase activity was estimated using a Dual-Luciferase Reporter assay system. *Renilla* luciferase activity was normalized against firefly luciferase activity.

### Western blotting

Total protein of transfected cells in logarithmic growth phase was extracted using RIPA lysis buffer. Then, 50 μg isolated protein were separated loaded onto a 10% gel, resolved using SDS-PAGE, transferred to PVDF membranes, and then blocked with 5% non-fat milk and probed with primary antibodies overnight at 4°C. Then, HRP-conjugated secondary antibodies were added at room temperature for 1 h. β-actin was used as a control. Blot bands were visualized using an ECL-PLUS kit.

### Statistical analysis

All statistical analyses in this project were performed using SPSS version 17.0. Experiments were repeated three times. All data are shown as mean ± standard deviation. A two-tailed Student’s t-test or paired t-test was performed to compare two groups. Analysis of variance was used for multiple comparisons. The association between circRNA_100859 expression levels and clinicopathological features was analyzed using Chi-square tests. Progression-free survival curves were performed using the Kaplan-Meier method. Prognostic analysis as conducted using univariate and multivariate Cox regression analysis. The correlation between circRNA_100859 and miR-217 and HIF-1α were evaluated using Pearson’s correlation analysis. *P*<0.05 was considered to indicate a statistically significant difference.

## Supplementary Material

Supplementary Figure 1
