# Supplementary material for: circRNA_100859 functions as an oncogene in colon cancer by sponging the miR-217-HIF-1α pathway
Source: Aging (Albany NY). 2020 Jul 8;12(13):13338–53. doi: 10.18632/aging.103438 (PMC7377858; doi:10.18632/aging.103438)
Supplement: Supplementary Figure 1 [file aging-12-103438-s001..pdf]

SUPPLEMENTARY FIGURE

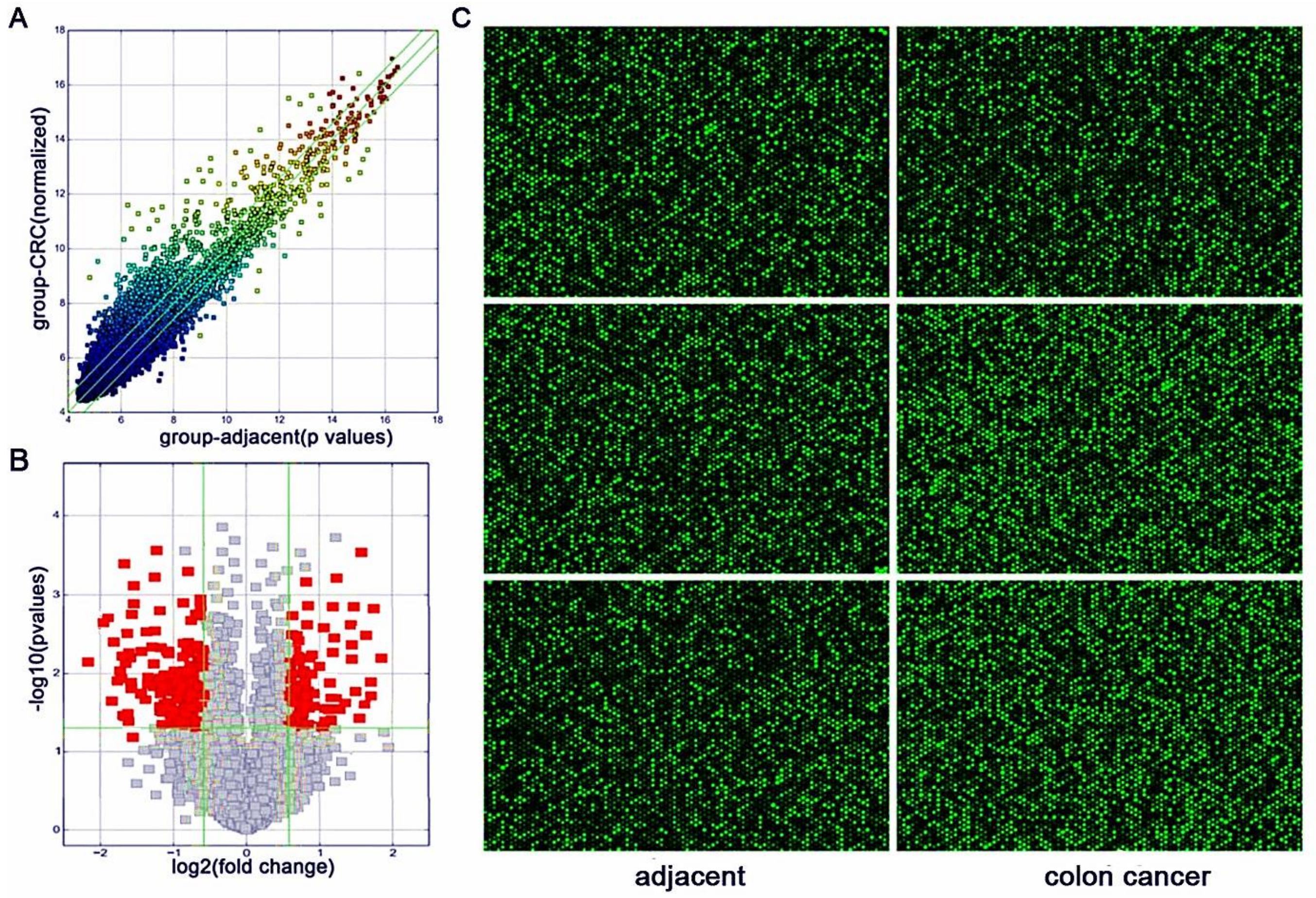

Supplemental Figure 1. Differential circRNA expression profiles of colon cancer were established successfully. (**A**) Volcano plots were constructed using fold change values and *P*-values. (**B**) Scatter plot illustrates the distribution of the data in circRNA profiles. The values of x and y axes in the scatter plot are the normalized signals of the samples (log2 scaled). (**C**) The circRNA microarray hybridization signals diagram of three pairs of samples.
